# Establishing the fundamentals for an elephant early warning and monitoring system

**DOI:** 10.1186/s13104-015-1370-y

**Published:** 2015-09-04

**Authors:** Matthias Zeppelzauer, Angela S. Stoeger

**Affiliations:** Media Computing Group, St. Pölten University of Applied Sciences, Matthias-Corvinus Strasse 15, 3100 St. Pölten, Austria; Department of Cognitive Biology, University of Vienna, Althanstrasse 14, 1090 Vienna, Austria

**Keywords:** Elephants, *Loxodonta africana*, Vocalizations, Acoustic monitoring, Automatic call detection, Call classification, Visual monitoring, Visual tracking, Human–elephant conflict

## Abstract

**Background:**

The decline of habitat for elephants due to expanding human activity is a serious conservation problem. This has continuously escalated the human–elephant conflict in Africa and Asia. Elephants make extensive use of powerful infrasonic calls (rumbles) that travel distances of up to several kilometers. This makes elephants well-suited for acoustic monitoring because it enables detecting elephants even if they are out of sight. In sight, their distinct visual appearance makes them a good candidate for visual monitoring. We provide an integrated overview of our interdisciplinary project that established the *scientific fundamentals* for a *future* early warning and monitoring system for humans who regularly experience serious conflict with elephants. We first draw the big picture of an early warning and monitoring system, then review the developed solutions for automatic acoustic and visual detection, discuss specific challenges and present open future work necessary to build a robust and reliable early warning and monitoring system that is able to operate in situ.

**Findings:**

We present a method for the automated detection of elephant rumbles that is robust to the diverse noise sources present in situ. We evaluated the method on an extensive set of audio data recorded under natural field conditions. Results show that the proposed method outperforms existing approaches and accurately detects elephant rumbles. Our visual detection method shows that tracking elephants in wildlife videos (of different sizes and postures) is feasible and particularly robust at near distances.

**Discussion:**

From our project results we draw a number of conclusions that are discussed and summarized. We clearly identified the most critical challenges and necessary improvements of the proposed detection methods and conclude that our findings have the potential to form the basis for a future automated early warning system for elephants. We discuss challenges that need to be solved and summarize open topics in the context of a future early warning and monitoring system. We conclude that a long-term evaluation of the presented methods in situ using *real*-*time* prototypes is the most important next step to transfer the developed methods into practical implementation.

## Background

Asian (*Elephas maximus*) and African (*Loxodonta africana* and *Loxodonta cyclotis*) elephants are the largest terrestrial herbivores, requiring considerable areas and a diversity of environments to forage [1, 2]. Therefore, their ranges are complex and not confined to officially designated protected areas. Due to the continuous spread of human settlements and cultivation areas and the hunting for ivory, elephants remain under threat from poaching [3, 4] and habitat loss. The resulting *human*–*elephant conflict* is a tremendous and constantly increasing problem. In particular, humans living near elephant habitats regularly get into serious conflict with migrating and crop-raiding elephants, which yields numerous victims on both sides each year [5, 6].

The alleviation of the human–elephant conflict as well as the fight against poaching requires autonomous systems that monitor elephant populations and their movements. Such a system might enable the realization of an *early warning system* for people living near elephant habitats to avoid the unexpected confrontation of humans and elephants. The foundation for such a system is techniques for the automatic detection and tracking of elephants. These tasks are challenging, especially when detection and tracking must be performed in a *non*-*invasive* manner to cover large areas and populations.

Different attempts towards the automatic detection of elephants have been made in the past. Techniques such as satellite tracking (efficient but also invasive) using global positioning system (GPS) [7], or light sensors with laser beams [8], and systems based on vibration sensors in the ground [9] are cost-intensive and might not scale to large populations. Today, non-invasive techniques such as acoustic and visual monitoring represent promising low-cost alternatives to sample populations and to obtain reliable estimates of species presence and, potentially, abundance [10, 11]. To approach the challenges of non-invasive monitoring, biologists and computer scientists have joined forces in an interdisciplinary research project to establish the *scientific foundations* for a *future non*-*invasive early warning and monitoring system* for elephants.

Elephants make extensive use of powerful low-frequency vocalizations termed “rumbles” with a mean fundamental frequency varying from 10 to about 30 Hz [12–15]. Elephant rumbles are sounds produced by passive vocal fold vibration similar to human speech [16]. The sound is filtered as it passes through the supra-laryngeal vocal tract, shaping the vocal tract resonances or formant frequencies [13, 17]. These low-frequency rumbles have been shown to travel distances of up to several kilometers [18]. This qualifies the elephant as a perfect model species for acoustic observation because elephants can be detected by their vocalizations even if they are out of sight [19, 20].

The calling rate of low-frequency elephant vocalizations was shown to be a useful index of elephant (*L. cyclotis*) numbers [19]. This demonstrates that acoustic surveying is a valuable tool for estimating elephant abundance as well as for detecting other vocal species; it can also help detect anthropogenic noises that may be associated with poaching [20–22]. Due to technological advances such as the development of autonomous and wireless recording devices with low energy consumption, the technological basis for acoustic recording in multiple locations over long time spans is established [11].

Elephants are the largest terrestrial herbivores and have a well-distinguishable visual appearance. Thus, visual information may be useful for the automated detection of elephants. In this project we investigated the suitability of visual cues for the automatic detection of elephants in wildlife video recordings and developed a first visual detection algorithm.

This project note summarizes results achieved during the project period (2011 until 2014), discusses the main challenges and contributions, and outlines future directions for the project team in order to transfer the promising fundamental results into practical implementation.

In this article we firstly draw the big picture of a non-invasive monitoring system and identify its necessary components. Secondly, we present our developed solutions for the automatic acoustic and visual detection of elephants—these represent the basis for all higher-level analyses performed by the envisioned system—and discuss their capabilities and limitations. As our research project is of an interdisciplinary nature, its contributions and findings have been published in a series of research articles across different highly specialized research communities. Thus, an additional contribution of this article is to provide an *integrated synopsis* of our findings for researchers of different communities and backgrounds (biologists, conservationists, computer scientists). Ultimately, we take the opportunity to present open (mostly interdisciplinary) tasks and challenges necessary for the establishment of an acoustic and visual early warning system to researchers from different domains to stimulate further joint research in this field.

### The future early warning and monitoring system: the big picture

**Fig. 1:**
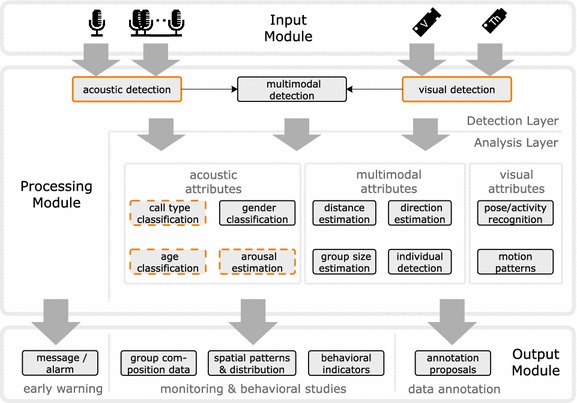
Overview of the envisioned elephant early warning and monitoring system. The first step of analysis is the detection of elephants either visually through video and thermal cameras or acoustically through a microphone (array). Different detection mechanisms may be combined in a multimodal approach. Automatically recognized detections can be directly input to an early warning systems or serve as a basis for higher-level tasks. The analysis layer contains the most important higher-level tasks in our context. Highlighted tasks have been investigated and automated in the course of the project

To give the reader an overview and an overall framework, we have sketched the architecture of the envisioned monitoring system in Fig. 1. The architecture serves as a frame for the identification of necessary components and open tasks and puts the different components in context. The sketched system consists of three modules: an *input module* with different sensors, a *processing module* where the sensor data is analyzed and an *output module* that provides analysis results. The sensor input can be highly multimodal, consisting of acoustic sensors (either individual microphones or microphone arrays to enable directional measurements) and different types of visual sensors (e.g. video cameras, “V” and thermal cameras, “Th”). The central processing module consists of a *detection layer*, where acoustic and visual detection (and potentially combined audio-visual detection) is performed. The detections form the basis for the generation of early warning messages and furthermore serve as an input to higher-level tasks in the overlying *analysis layer.* Higher-level tasks comprise aspects that might be relevant for researchers, for example call type classification, age and gender identification, arousal or group size estimation. Additionally, methods from the analysis layer may assist biologists in the annotation of field data based on the proposal of annotation tags (semi-automatic annotation).

In the following we focus on presenting research and results on the acoustic and visual detection of elephants (detection layer). We start with the description of data collection and annotation that was necessary to build evaluation datasets for the developed detectors (Section “Data Collection and Annotation*”*). Next, we present the methods developed during the projects and the corresponding findings (Section “Methods and Findings”). Finally, we discuss remaining challenges and necessary future work in the context of automatic elephant detection (Section “Discussion”).

## Data collection and annotation

The collection of acoustic and visual data under natural field conditions is one important prerequisite for the development of robust elephant detection methods. A second prerequisite is the precise annotation of the recorded material (e.g. temporal annotation of calls in acoustic recordings and spatio-temporal annotation of individuals in video).

### Data collection

We collected elephant vocalizations in two different locations in South Africa. The aim was to obtain (1) high-quality recordings and (2) data heterogeneity concerning environmental conditions, recording distance, call types, contexts, age groups and gender. We performed stereo-recordings with a directional AKG microphone (AKG 480 B CK 69, frequency response 8–20,000 Hz ± 0.9 dB) and an omni-directional Neumann microphone (KM 183) modified for recording frequencies below 20 Hz (flat recording down to 5 Hz) connected to a 722 Sound Device HDD recorder. Concurrent video recordings were performed using a Sony DHC-SD909 camcorder in HD quality to supplement field notes, to assist the subsequent acoustic annotation, and to enable the development of automated visual detection methods. Acoustic and visual recordings were performed at two locations:

1. Recordings were collected from three female and two male African elephants aged between 9 and 17 years located at Adventures with Elephants, Bela Bela, South Africa. The elephants were fully habituated to human presence and live in a 300 ha savannah reserve. This enabled us to capture high-quality data under controlled recording settings within the natural habitat of African elephants. For detailed descriptions about data collection we refer to [23, 24].
2. Free-ranging elephants were recorded at the Addo Elephant National Park, Eastern Cape Province, South Africa. The South African National Park research committee has approved these non-invasive recording activities. In 2008, the elephant population numbered 481 individuals with an annual rate of increase of 5.81 % (between 1976 and 2002), split into seven family groups [25]. Data were collected out of a vehicle (with the equipment fixed on a custom-built tripod at the door of the car) during June and July 2011 and July and August 2012, yielding 101.4 h of recordings. The focus was on collecting vocalizations of individuals in differing situations and activities (such as feeding/browsing, at the waterhole and locomotion), and of various age classes and gender. The identity of the vocalizing elephant was sometimes determined for adult females, but could rarely be retrieved for calves and infants. Most recorded calls originate from adult females, but we have also been able to capture sufficient vocalizations from infant, calf and juvenile individuals. Only few vocalizations of adult males could be recorded. For more details on data collection, refer to [26].

The acoustic recordings were—whenever possible—accompanied by visual recordings from a camera (Sony DHC-SD909 camcorder) fixed on a tripod on the car. We took close-up videos of individuals to enable identification of the callers later in the lab, but also performed continuous recordings of particular situations. Here we avoided zooming to simulate the recording settings of a stationary surveillance camera. Pan and tilt movements were conducted to observe larger areas and to follow elephant movements. During the field sessions at the Addo Elephant National Park a total of 22 h of video material (1920 × 1080 pixels at 25 frames per second) was recorded, corresponding to 2 million frames and 150 GB of unannotated data.

### Data annotation

**Fig. 2:**
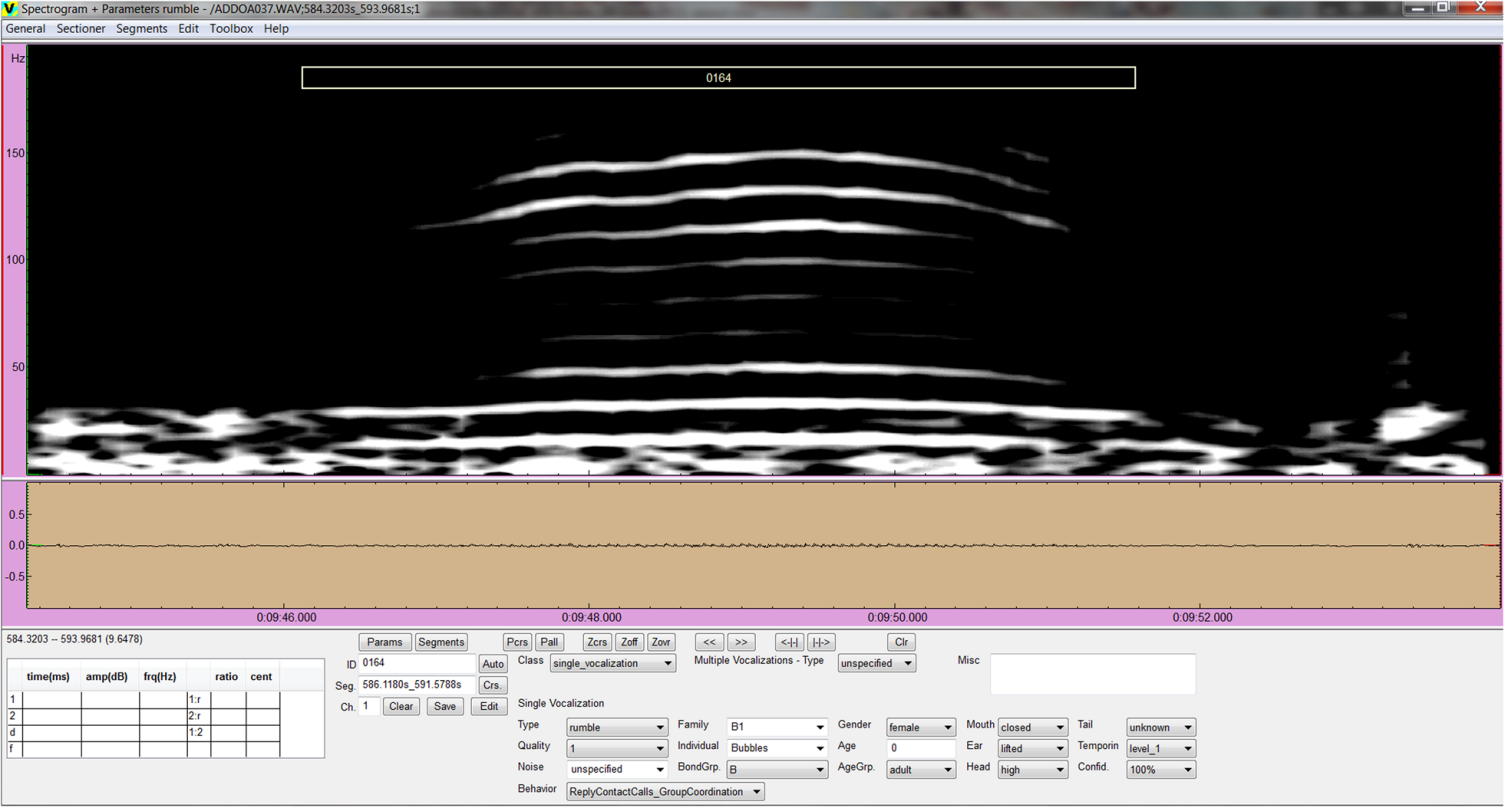
The interface for manual annotation. The interface shows the spectrogram of a recording and numerous annotation fields for the detailed description of all observed vocalizations

The acoustic recordings were annotated using the acoustic analysis tool STX from the Acoustic Research Institute at the Austrian Academy of Science. We customized the tool to enable the detailed annotation of calls and call types together with their social context and information about the caller (if known). Each vocalization was identified based on field notes, videos, by listening, and by examining the spectrogram. We also annotated overlapping calls, call combinations and choruses. The start and end cues of each vocalization were tagged and the corresponding annotations were added. Figure 2 shows the annotation interface on a recording from the Addo Elephant National Park.

The annotations were stored in XML format. We developed an interface that imports the complete annotations into MATLAB to generate ground truth data that are suitable for the automated evaluation of call detection, call segmentation and classification methods.

We annotated 2199 vocalizations (of different call types) from free-ranging elephants at the Addo Elephant National Park, and 681 vocalizations recorded at Bela Bela. 633 of these vocalizations were rumbles (see Table 1 for a detailed overview on the number of calls recorded for each call type during each field session). The complete and precise annotation of the recorded data formed the basis for objectively evaluating the automatic analysis methods developed in the project.

**Table 1:** List of available data on elephant vocalizations

Call type	N calls recorded at Bela Bela 2011	N calls recorded at Addo 2011	N calls recorded at Addo 2012
Rumbles	633	925	529
Barks	1	15	1
Noisy roars	18	41	19
Tonal roars	0	34	23
Mixed roars	0	56	20
Trumpet	15	166	137
Snort	3	103	87
Grunt	0	8	7
Unknown	11	22	6
*Sum*	*681*	*1370*	*829*

This table lists the number (N) of calls that have been recorded and annotated for each field session

**Fig. 3:**
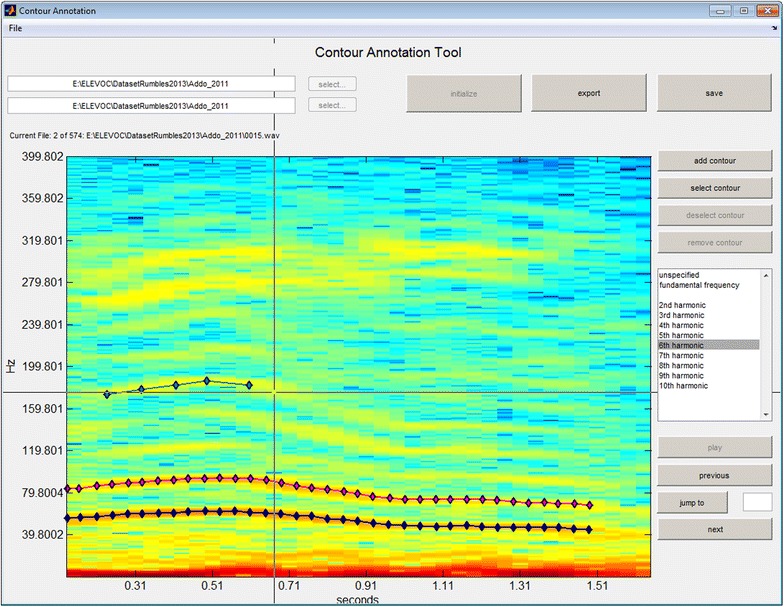
The developed semi-automatic sound annotation tool. The user can annotate and label frequency tracks. Features are then computed automatically from the contours

To develop automated acoustic feature extraction methods, we identified the characteristic parameters of different call types, e.g. observed frequency range, the frequency progression over time, the duration, harmonic structure, and the range of formant frequencies. As the automatic detection of fundamental frequency and formant frequencies is often error prone in noisy (real-world) recordings and partially requires manually tuning the processing parameters, we developed an annotation tool during the project to measure basic acoustic parameters semi-automatically. This tool enables manually labeling frequency contours in the spectrogram of a given call and thus enables describing the spectral and temporal characteristics of source- and filter-related attributes in noisy and acoustically interfered vocalizations. To capture the variance of observed parameters under real-world conditions, we employed the data recorded in Addo Elephant National Park and at Bela Bela [23, 26]. Figure 3 shows the annotation interface with some annotated frequency contours.

The automatic evaluation of our acoustic detection algorithms required a comprehensive dataset of recordings that originated from one geographical location. The data recorded at Bela Bela were well-suited for this purpose. The dataset captured recordings from different recording sessions, at different times of the day and with varying environmental noise levels. With a total of 6 h and 635 distinct vocalizations, the set significantly surpassed the datasets of other related works in length [27] and number of vocalizations [28].

Similarly, for the evaluation of visual detectors it was necessary to compile a reference dataset from the collected video material. We selected a representative subset of the entire video collection to speed up subsequent annotation and further processing. During selection we rejected sequences that were too similar to already selected ones to increase the heterogeneity in the dataset. The selected sequences contained elephants (groups and individual elephants) of different sizes from two distance categories (far distance and near distance). Elephants were visible in arbitrary poses and ages, performing different activities such as eating, drinking, running, and different bonding behaviors. The sequences showed different locations, such as elephants at a water hole, elephants passing a trail, and highly occluded elephants in bushes. Selected sequences reflected different times of the day and showed different lighting and weather conditions. Furthermore, recording settings varied across the sequences (e.g. from almost static camera mounted on a tripod to shaking handheld camera). Additionally, the subset contained sequences with no elephants at all and sequences where elephants entered and left the scene. The heterogeneous settings of the selected scenes were necessary to reflect the broad variability of real-world conditions to enable the development of robust visual detectors that can cope with a variety of different situations.

After creation of the video set, each individual elephant was tagged precisely in space and time, i.e. each pixel in a frame that showed a (possibly incomplete) body part of an elephant was tagged. To create a spatiotemporal ground truth for each sequence in our video dataset, we first extracted all individual frames of the sequence. Next, the first frame was semi-automatically labeled by an image processing tool such as Gimp or Adobe Photoshop. As subsequent frames were usually highly similar to the previous ones, we used the initial labeling and iteratively adapted it to the subsequent frames. The entire ground truth generated covers 715 frames and 1751 manually labeled segments covering elephants. The accurate spatio-temporal labeling represented an important prerequisite for the objective and precise evaluation of our visual detection method.

## Methods and findings

In the following we present our approaches for the acoustic and visual detection of elephants. For both approaches we first provide background information, sketch the developed method, and finally present and discuss our evaluation results.

### Acoustic detection of elephants

#### Background

The major challenges in the robust detection of elephant rumbles are (1) the large number of natural and environmental influences (e.g. wind, rain, other animals, engine sounds) and (2) the broad natural variability of elephant rumbles. Structural variations of elephant calls arise due to differences in vocal production, social contexts, states of arousal and motivation, hormonal states and maturational effects. In addition, sounds are altered with distance and environmental influences [18, 29]. What further increases the complexity is that elephant calls are rare events in long-term environmental recordings (e.g. elephant calls represent much less that 1 % of our recordings) and show erratic occurrence patterns.

The low-frequency rumble is the most studied elephant vocalization with regard to function and production [e.g. 29–38]. The rich knowledge gained in these investigations (prior to and also during the project) represents a solid basis for developing an automated acoustic elephant detection method.

Most research on acoustic (and partly automated) analysis of elephant vocalizations has addressed highly selective tasks such as the vocal identification of individual elephants [29, 30, 33] or the analysis of particular call types [34, 35] and subtypes [23, 26, 32, 36–39] from manually selected and *pre*-*segmented* calls. The automated detection of elephant vocalizations is much more complex because the calls must be localized and segmented out of a complex soundscape.

To date, only little research has been performed on the automated detection of elephants. Venter and Hanekom [28] detected elephants based on their rumbles in wildlife recordings by extracting the characteristic fundamental frequency of rumbles using sub-band pitch estimation. They defined those audio segments as rumbles in which a pitch (in the typical frequency range of rumbles) can be tracked robustly for a certain amount of time. A similar approach also relying on pitch extraction has recently been proposed by Prince and Sugumar [40] for elephant detection. Wijayakulasooriya [27] proposed an alternative rumble detection method that employs the shape of formant frequency tracks as a clue. The basic assumption of the latter method is that the first and second formants are nearly stationary during a rumble. Thus, detecting audio sequences with stationary formant frequencies in the frequency range of rumbles should provide clues for their acoustic detection.

We have investigated related approaches in detail in [41] and draw the following conclusions: (1) the detection of pitch and formant frequencies is itself a complex audio analysis task that often fails in the presence of noise and requires prior knowledge about processing parameters that are often not available or not constant for unconstrained wildlife recordings; (2) pitch and formant frequencies are often masked by low-frequency noise and thus hardly visible in the spectrum, which puts their usability for automated detection under real-life settings into question.

Our approach avoids the explicit detection of pitch and formant frequencies and instead takes the entire spectral distribution (including the harmonic structure and implicitly also pitch and formant frequencies) into account. This provides additional important information for detection. Additionally, we apply machine learning to model representative acoustic patterns of elephant vocalizations directly from the data instead of assuming characteristics (e.g. particular formant contour shapes) to apply.

#### Method

**Fig. 4:**
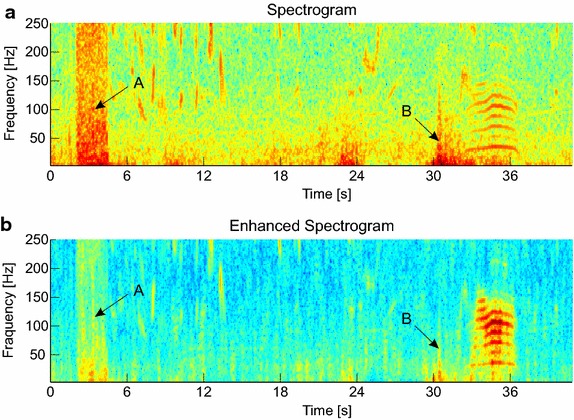
The effect of sound enhancement. **a** The input spectrogram with a rumble at 35 s and several noise sources, e.g. broadband noise (label “*A*” and label “*B*”); **b** The enhanced spectrogram after signal enhancement. The energy of the spectro-temporal patterns related to the rumble is increased while the signal energy of the noise sources is attenuated (best viewed in color)

As mentioned above, the major challenge in automatically analyzing vocalizations from field recordings is the broad range of uncontrollable noise sources in the environment. To account for these influences we have developed a method for noise reduction that attenuates noise from the environment while retaining the distinct frequency patterns originating from elephant rumbles. The method applies image processing techniques on the spectrogram to enhance well-localized spectro-temporal patterns such as frequency contours. At the same time our noise reduction technique attenuates the spectral energy of weakly localized components, for example narrow- and broadband noise from wind and rain [24, 41]. This improves the signal-to-noise ratio of recordings and thus the “visibility” of rumbles in the spectrogram. Figure 4 illustrates the effect of sound enhancement on the spectrogram.

The signal enhancement represents only the first step of our acoustic detection method. After enhancing the spectrogram, we compute short-time spectral features (Greenwood-frequency cepstral coefficients, GFCC) to parameterize the individual frames of the recorded signal. The employed frame size is 300 ms with an overlap of 2/3. GFCC are an adaptation of the more popular MFCCs (Mel-Frequency Cepstral Coefficients) from speech recognition, which explicitly model the critical bands of the elephants’ hearing system [42]. To obtain a more expressive (longer-term) audio description, we aggregate the GFCC feature vectors of subsequent audio frames by computing their mean and average.

The mean and average vectors are finally concatenated and used to train a classifier. In our experiments a linear SVM classifier (Support Vector Machine, [43]) showed good discrimination ability between background sounds and elephant rumbles. The SVM was trained from a set of positive and negative sound samples (the training set). Once trained, the SVM was applied to new, previously unseen field recordings from the test set to evaluate the detection performance. We refer the interested reader to [24] and [41] for more details on the implementation of the detection method.

#### Results

From the captured wildlife recordings, we observe that many noise sources corrupt the frequency band where rumbles reside. This often masks the characteristic fundamental frequency and parts of the harmonic structure. The most interfering noise sources are wind and rain, as well as engine sounds of cars and airplanes. First qualitative evaluations on a set of example sounds indicate that the proposed signal enhancement technique improves the signal-to-noise ratio of the rumbles in noisy environments. Figure 4 illustrates the effect of sound enhancement on an example sound that contains numerous noise sources.

We quantitatively evaluate our method on the dataset described in Section “Data Annotation”. For this purpose, the dataset is split into a training and test. The training set contains 63 randomly selected rumbles (10 % of all rumbles in the dataset) and 30 sequences without rumbles. The remaining data are used as test set. We perform automatic detection on the test set and compute the detection rate as well as the false-positive rate. The quantitative results are shown in Table 2.

**Table 2:** Automatic detection results for different related and the proposed method

Method	Detection rate (%)	False-positive rate (%)
Hao et al. [44]	78.6	78.6
Venter and Hanekom [28]	85.7*	14.2*
Proposed method without signal enhancement	88.2	24.4
Proposed method with signal enhancement	88.2	13.7

Signal enhancement strongly influences detection performance, especially the false-positive rate

* Results by Venter and Hanekom [28] are not directly comparable as they were obtained for a different (smaller) dataset with fewer rumbles

We further compare our results with those of related work. The results reported by Wijayakulasooriya [27] were obtained from a dataset encompassing a few minutes (less than 15 min) of recordings and are thus not directly comparable. The dataset used by Venter and Hanekom [28] consists of a total of 4 h of recordings with 28 elephant rumbles. They achieved a detection rate of 85.7 % with a 14.2 % false-positive rate.

In addition we applied the method by Hao et al. [44] on our dataset. The method is based on spectral template matching and was proposed as a general-purpose approach for the detection of animal sounds. While the detection rate is promising, the false positive rate of 78.6 % shows that a simple template matching is unable to distinguish rumbles robustly in noisy conditions. Our developed baseline system without signal enhancement yields a detection rate of 88.2 % at a false positive rate of 24.4 %. The same system with signal enhancement achieves a similar detection rate but nearly halves the false positive rate to 13.7 %. This demonstrates that signal enhancement strongly improves detection performance. Our proposed method thus outperforms related ones in detection rate and false-positive rate.

A detailed investigation of false detections [24] shows that the most interfering noise sources are airplanes and car engines. This is because they have fundamental frequencies and harmonic structures similar to elephant rumbles and are thus difficult to distinguish. The inspection further reveals that some “false detections” actually represent elephant rumbles that were for some reason not annotated, for example because they were combined or partly masked by other calls or because they were overlooked; see [24] for details. These results highlight the strong potential of the developed method.

### Visual detection of elephants

Elephant calls are rare events and, thus, acoustic methods alone might not be a sufficient solution for a reliable detection in all contexts. A promising candidate to complement acoustic detection at certain locations might be the visual detection of elephants, due to their body size and salient appearance.

#### Background

At the time the project was started (in 2011) the automatic visual detection of elephants had not been studied yet. The detection of animals was addressed mostly in the course of object recognition benchmarks [45] based on user-generated images of e.g. cats and dogs. The detection of animals from wildlife data was addressed by only a few authors, for example to detect zebras, giraffes, and tigers [46–48]. Related literature shows that visual analysis has mostly focused on animals with a well-textured skin because the rich texture facilitates visual.

The visual detection of animals such as elephants without a distinct skin texture poses additional challenges to visual analysis. Attributes such as color, shape, and motion need to be exploited, but plants and trees often occlude elephants, revealing only body parts rather than the whole elephant. Additionally, elephants appear in different postures and sizes, as well as in groups and as individuals. Thus the typical shape of an elephant as well as its size are generally not useful clues for their visual detection. Similarly, motion is a questionable visual clue for detection, as elephants often move slowly or rest for lengthier times.

During our project we investigated the capabilities of visual elephant detection and developed a first method for visually detecting and tracking elephants in wildlife video recordings. The method exploits color clues as well as temporal clues (continuity) and is applicable to videos showing complex scenes where vegetation introduces extensive occlusions [49].

#### Method

We proposed a method for elephant detection that exploits the color information contained in a given scene. In a first step, we learned a color model of elephant skin from a set of annotated training images. All pixels associated to body parts of elephants were used to model the skin. The remaining colors surrounding the elephants were employed to build a color model of the background. A binary Support Vector Machine (SVM, [43]) was trained from the color data. The trained SVM was later used to predict whether a given color more likely represents elephant skin or background.

The classification of each individual pixel in a video is a computationally demanding task. To accelerate the process, we apply image segmentation to the individual frames of the video and then perform detection based on image segments. Segmentation is performed based on Meanshift and takes color and edge information into account to find visually consistent segments in the input frame [50]. The parameters of the algorithm are tuned such that an over-segmentation of the image is obtained, i.e. an object is split into multiple segments rather than merged with another similarly looking object. We account for over-segmented objects during temporal analysis in a subsequent processing step.

Given a segmented input frame, we apply the trained color model to all segments to identify areas in the video frames that are likely to correspond to elephant skin. Note that this works even in the case of heavy occlusion, as no assumptions about elephant shape and size are made. The result is a set of image segments (candidate detections) for a given video frame.

**Fig. 5:**
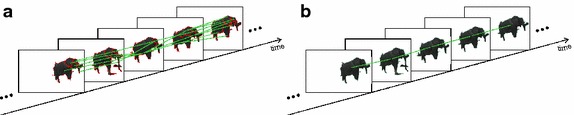
Spatio-temporal refinement for candidate detections. **a** Spatially consistent candidate detections are tracked over time. Relations between these detections can be used to spatially merge candidate detections. The result **b** is more robust detections that are consistent over time

In the next step, we track the candidate detections over time and merge temporally coherent detections in consecutive frames that are likely to belong to the same object (see Fig. 5 for an illustration). Details on tracking and merging can be found in [49]. The temporal tracking of candidate detections yields spatio-temporally consistent detections that provide additional (stronger) clues for elephant detection and the rejection of false detections. We apply a simple rule to refine the candidate detections: segments that can be tracked consistently over a large number of frames at a similar location are more likely to represent an elephant than a segment that suddenly disappears or that changes location abruptly (which is often the case for false positive detections). The temporal continuity analysis helps to identify false detections and noise and improves the detection quality of the method. As a by-product of temporal analysis we obtain all information necessary to track the detected elephants in image space over time. We refer the interested reader to [49] for more details on the developed method.

#### Results

To evaluate the visual detector, we employed a heterogeneous set of video recordings from Addo Elephant National Park described in Section “Data Collection and Annotation”. As the video sequences in our dataset show elephants at different distances, we evaluated our method in two different settings: first, for near distances (up to approx. 50 m) and, second, for far distances (above 50 m). The detections generated by our method were compared to the previously generated ground-truth to compute performance measures such as detection rate and false positive rate.

**Fig. 6:**
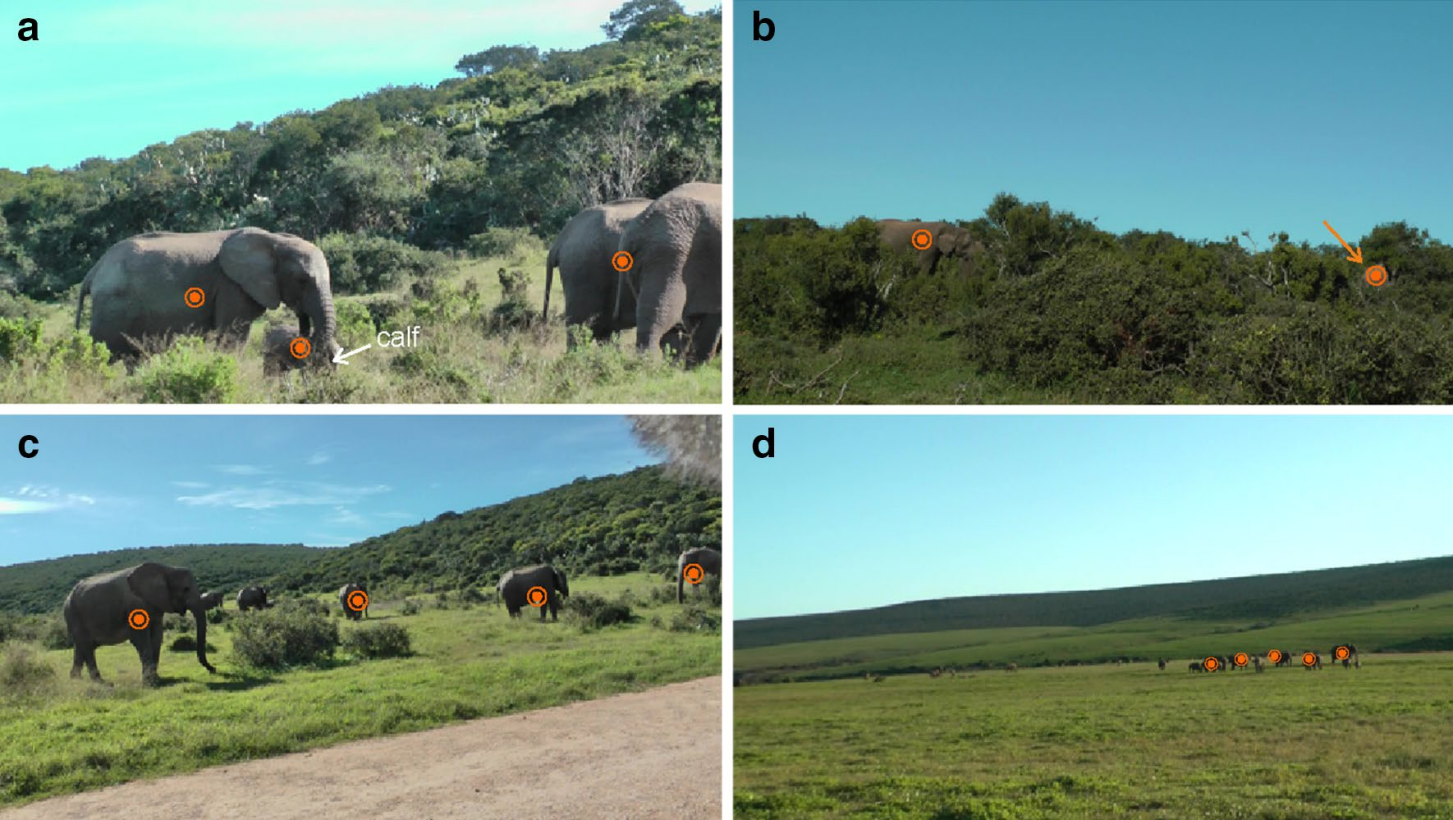
Results for visual elephant detection. Automatically detected elephants at near distances (**a**, **b**, **c**) and at far distance (**d**) [49]

Figure 6 shows qualitative results for both scenarios. Figure 6a demonstrates that elephants of different sizes can be detected using the same detection method. The depicted frame shows three adults and one calf. The adults and the calf are detected successfully. The two adults on the right side are reported as one detection by our method. This is due to the large overlap of the two individuals. We further observe that even elephants that are highly occluded by vegetation as shown in Fig. 6b can be detected successfully. This scene further demonstrates why shape is in many situations not a useful clue for detection, as only individual body parts of the elephants are visible.

The results of our quantitative evaluation on the entire video dataset are summarized in Table 3. For elephants at near distances we obtained a detection rate of 91.7 % at a false positive rate of only 2.5 %. This means that most elephants are detected successfully (only 8.3 % are missed) and the rate of false detections is quite low (only every 40th detection is actually not an elephant).

**Table 3:** Quantitative results for visual elephant detection at near and far distances

Range	Detection rate (%)	False-positive rate (%)
Near	91.7	2.5
Far	88.0	39.0

The overall detection performance is especially high at near distances while for far distances the problem becomes increasingly complex, which is reflected by the high false-positive rate

With larger distance the detection becomes increasingly difficult. The small area covered by the elephants in the image plane requires a much finer image segmentation of the input images. This results in a stronger over-segmentation of the image and thus a large number of small image patches. The small patches exhibit fewer distinctive visual features useful for automated detection than larger segments, which impedes automated detection and results in more detection errors for far-distant elephants. Our experiments clearly show the effect of the smaller object size on the detection rate and false positive rate (Table 3).

Our experiments indicate that a robust detection and tracking of elephants in wildlife videos is feasible and particularly successful at near distances. We are able, in video, to detect and track elephants of different sizes and postures and in different activities. Our method is robust to occlusions and to the complex structure of wildlife scenes. Since the color model for elephant skin and background can be learned from training data, the method can easily be adapted to different backgrounds and lighting conditions.

## Discussion

The overall goal of the project was to develop automatic analysis techniques to establish the foundations for a future detection and monitoring system for free-ranging elephants. As acoustic detection might not be sufficient in certain situations, we further investigated the feasibility of elephant detection in the visual domain. The developed acoustic and visual detection methods represent two basic building blocks for a future early warning and monitoring system illustrated in Fig. 1 and thus represent a first step towards this overall goal. In the following we draw conclusions from the performed work and summarize challenges and open topics in the context of automatic audio-visual detection.

From our work and experiments on the acoustic detection of elephant rumbles, we learn that signal enhancement is the key to robust detection. While powerful features exist in the literature from related domains such as speech recognition, the role of signal enhancement has been underestimated so far for detecting elephant vocalizations. One potential reason for this is that sound enhancement was not necessary in many studies because the employed audio recordings were performed under controlled settings with a high signal-to-noise ratio. An example is the work in [42] where the microphones were attached directly to (captive) elephants using special collars. Our investigations have shown that unconstrained audio recordings from larger distances, however, call for a suitable signal enhancement that improves the signal-to-noise ratio to achieve robust detection.

We further conclude that an acoustic detector must be highly adaptable to the environment it is operating in and to the observed population. Different environments exhibit different acoustic characteristics and soundscapes, e.g. due to nearby roads and railways. Additionally, there are species-specific differences in the vocal repertoire of elephants [15]. In Asian elephants, the rumble dominates the repertoire less than in the African species. The most remarkable difference is the highly pitched (repetitive) vocalizations (chirps or squeaks and squeals), which are completely missing in the African species. Moreover, elephants have vocal learning capabilities [51, 52] which further lead to differentiation of populations. A future acoustic detection system must be able to adapt to the environmental settings and the observed population. To take environmental variations into account we use powerful machine learning techniques to learn models of elephant calls as well as the background soundscape. An important prerequisite for machine learning approaches is the collection of field recordings (together with detailed annotations) that reflect the real-world complexity of the surrounding soundscape in which the detector should operate in the future as well as the rich variability of observed elephant calls. Thus, given representative training data, an adaptation to a different species (e.g. Asian elephants) is possible.

The developed acoustic detector represents a first prototype that was trained and evaluated with field data in the laboratory. For future testing and subsequent continuous operation of the detector in the field, a number of research and engineering tasks remain that we compactly summarize in the following:

- Major sources for false positive detection are engine sounds of cars and airplanes. As both sound sources cannot be isolated from elephant territory, the detector must be made robust against these sound sources. Our investigations show that the harmonic structure of engine sounds is too similar to separate these sounds from elephant rumbles. We observe, however, a different temporal structure, i.e. a longer duration, for engine sounds than for elephant rumbles. Thus, a future direction of research is to extend the detector with a multiscale analysis along the temporal dimension to separate sound events with different temporal scale.
- Another important aspect is that aside from elephant rumbles, a number of other elephant calls occur, most notably trumpets, snorts, and roars (see also Table 1). Existing acoustic detectors concentrate on the rumble only. For an early warning system, however, all call types should be taken into account. As each call type is characterized by different spectro-temporal patterns, a single detector is insufficient to recognize all call types. Thus, we suggest to train individual detectors for each call type separately and to combine them into a detector ensemble. Such an ensemble should enable the recognition of many different call types and potentially improve detection performance by exploiting dependencies (correlations) between the detector outputs.
- The training of our detector requires a certain amount of field data (calls of interest and background sounds). The collection of such field data is time-consuming and expensive. To reduce the effort, we propose using semi-supervised learning strategies in the future to incrementally train the detectors in the field. In semi-supervised learning the detector starts with a small amount of initial training data. In a first online learning phase, for every detected vocalization the system asks the user (or multiple users) for feedback on whether or not this detection is correct. From the provided user feedback the detector incrementally refines its model. This approach can strongly reduce the effort for data collection and annotation and at the same time the detector can optimally adapt to its operating environment.
- An important open task for future research (and engineering) is implementing an autonomous hardware platform capable of running the detector (or the detector ensemble) in real-time and acting as an early warning system. Such a hardware platform must be computationally strong enough to perform multiple spectral analyses and classifications in real-time and, at the same time, energy efficient enough to run continuously supplied by a battery that is continuously recharged by, for example, solar energy. Additionally the unit must be connected to some network, either a cellular network (to issue alarms) or in the absence of such a network to some signaling infrastructure such as warning lights or sirens that alert nearby people [53]. Finally, the device and the microphone(s) must be adequately protected from the wind and rain.

A second outcome of the project is a first algorithm for the *visual detection* of elephants that is applicable to unconstrained wildlife video. From our investigations we learned that the complexity of visual detection is high because the target to detect is particularly rare and the uncontrollable environment poses numerous challenges including occlusions by vegetation, low contrast due to shadows, and poor illumination due to front lighting. Our experiments show, however, that for limited distances, elephants can be reliably detected.

A crucial factor for robust visual detection is to minimize assumptions about the environment and target objects (e.g. the number of elephants, their posture and orientation to the camera, the background). We achieve this goal by employing a color model of elephant skin, which can be trained adaptively depending on the operating site and the illumination situation. Similarly to acoustic detection, the adaptation to the operating environment by learned models is important for robust detection.

The temporal processing in our method enables tracking individual detections over time in the video. This makes the approach a sound basis for higher-level analysis tasks, from the automated estimation of group sizes to the automated recognition of different activities and potentially behaviors.

From our investigations we conclude that visual detection is especially useful along corridors and at frequently attended sites such as waterholes. In such places cameras can be placed close to the elephants, enabling robust visual detection. The computational complexity of the visual detection algorithms developed during the project, however, significantly exceeds that of acoustic detection and poses numerous challenges to the further implementation of a visual monitoring system:

- In a first step, the developed algorithms must be accelerated to enable real-time processing. The computationally most demanding processing steps in our detection pipeline are image segmentation and motion analysis (optical flow computation). For both tasks, efficient algorithms have been proposed recently that enable real-time processing on modern graphic cards [54, 55].
- The developed algorithm is able to operate on video from moving and also shaking (handheld) cameras. For many monitoring settings this is not necessary because the camera is fixed and does not change the viewing perspective. For such a setting, we recommend to learn background models of the environment [56] that enable the separation of foreground and background. Different background models can be learned for different times of the day to account for illumination differences. Once the respective model has detected the background, only the foreground must be processed, which strongly reduces the computational demands.
- In the presence of limited processing power, we further suggest the development of image-based rather than video-based detectors. Although our experiments in [49] have shown that temporal continuity from the video stream significantly improves the detection reliability, we believe that in a setting with a static camera and learned background models the absence of temporal information can be compensated. Such a system can be implemented on low-cost hardware platforms such as Raspberry PI [57] in combination with an onboard camera that is triggered at regular intervals. The pre-trained background models can be applied in real-time on the platform and only the detected foreground objects (which usually make up only a small fraction of the entire image) must be analyzed further (either directly on the hardware platform or on a base station connected through available network infrastructure).

In the course of our project, we have investigated the detection of elephants in the acoustic and visual domain. We believe that detectors from both domains have the potential to compensate each other’s weaknesses. Acoustic detectors work well even when the elephants are out of sight, enable detecting elephants at long distances and can operate during day and night. Visual detectors in contrast do not require the elephants to vocalize and can permanently monitor corridors and places of interest during the day.

The future establishment of a reliable early monitoring system will require combining multiple complementary modalities to compensate the weaknesses of individual detectors and thus to improve the overall detection quality. In addition to acoustic and visual detectors, we propose to complement a future monitoring system with thermal cameras that might provide additional useful information for elephant detection. Thermal cameras enable visual detection at night and may improve detection performance of visual detectors during the day by adding an additional complementary temperature channel to the color information. For the realization of the envisioned early warning and monitoring system as depicted in Fig. 1 at the beginning of this article, we believe that only a combination of multiple orthogonal sensors will lead to satisfactory results.

In summary, the scientific foundation for an elephant acoustic detection and early warning system for humans living near elephant habitats is promising. Numerous requests from conservationists in Asia and Africa show that such a system is urgently needed, yet funding to further improve the detector and evaluate it in the field is required in order to transfer the promising research results into practical implementation. This would be an important step forward in mitigating the human–elephant conflict.
